# Faster DNA Repair of Ultraviolet-Induced Cyclobutane Pyrimidine Dimers and Lower Sensitivity to Apoptosis in Human Corneal Epithelial Cells than in Epidermal Keratinocytes

**DOI:** 10.1371/journal.pone.0162212

**Published:** 2016-09-09

**Authors:** Justin D. Mallet, Marie M. Dorr, Marie-Catherine Drigeard Desgarnier, Nathalie Bastien, Sébastien P. Gendron, Patrick J. Rochette

**Affiliations:** 1 Axe Médecine Régénératrice, Centre de Recherche du CHU de Québec–Université Laval, Hôpital du Saint-Sacrement, Québec City, Québec, Canada; 2 Centre de Recherche en Organogénèse Expérimentale de l'Université Laval/LOEX, Université Laval, Québec City, Québec, Canada; 3 Département d’Ophtalmologie et ORL—chirurgie cervico-faciale, Université Laval, Québec City, Québec, Canada; University of South Alabama Mitchell Cancer Institute, UNITED STATES

## Abstract

Absorption of UV rays by DNA generates the formation of mutagenic cyclobutane pyrimidine dimers (CPD) and pyrimidine (6–4) pyrimidone photoproducts (6-4PP). These damages are the major cause of skin cancer because in turn, they can lead to signature UV mutations. The eye is exposed to UV light, but the cornea is orders of magnitude less prone to UV-induced cancer. In an attempt to shed light on this paradox, we compared cells of the corneal epithelium and the epidermis for UVB-induced DNA damage frequency, repair and cell death sensitivity. We found similar CPD levels but a 4-time faster UVB-induced CPD, but not 6-4PP, repair and lower UV-induced apoptosis sensitivity in corneal epithelial cells than epidermal. We then investigated levels of DDB2, a UV-induced DNA damage recognition protein mostly impacting CPD repair, XPC, essential for the repair of both CPD and 6-4PP and p53 a protein upstream of the genotoxic stress response. We found more DDB2, XPC and p53 in corneal epithelial cells than in epidermal cells. According to our results analyzing the protein stability of DDB2 and XPC, the higher level of DDB2 and XPC in corneal epithelial cells is most likely due to an increased stability of the protein. Taken together, our results show that corneal epithelial cells have a better efficiency to repair UV-induced mutagenic CPD. On the other hand, they are less prone to UV-induced apoptosis, which could be related to the fact that since the repair is more efficient in the HCEC, the need to eliminate highly damaged cells by apoptosis is reduced.

## Introduction

The role of UV exposure in the prevalence of sun-related cancers is well documented [1–5]. The genotoxic effect of UV wavelengths lies in their capacity to be directly absorbed by DNA and to covalently bind adjacent pyrimidines, forming the cyclobutane pyrimidine dimers (CPD) and the pyrimidine (6–4) pyrimidone photoproducts (6-4PP) [6–8]. CPD are the most pro-mutagenic UV-induced DNA adducts [6–9] and are responsible for C→T and CC→TT transition mutations at dipyrimidinic sites, the signature mutations induced by UV light [10–14].

In skin, UV rays can transform the three cell types of the epidermis and therefore lead to the three known types of skin cancer: basal cell carcinoma, squamous cell carcinoma and melanoma [9, 15–19]. Solar exposure also causes many pathologies of the ocular surface. It is well documented that exposure to solar UV light is a proven risk factor in the incidence of pterygium, ocular surface squamous neoplasia (OSSN), climatic droplet keratopathy and actinic conjunctivitis [20–24]. OSSN arise mainly in the limbal region, the stem cell compartment of the cornea, but can also arise from the conjunctival or corneal region. However, the implication of UV-light in the corneal OSSN remains controversial. Indeed, the incidence of OSSN is 0.3 per million in the US [24] and 86% does not involve the cornea [25]. In comparison, non-melanoma skin cancer incidence is over 15 000 per million in the US [26]. Although the cornea shares functional and structural similarities with the skin, there is a clear difference in sensitivity to UV-induced neoplasia between corneal epithelial and epidermal cells.

The ocular region is undeniably exposed to UV light, as a proof 5 to 10% of all skin cancers occur in the eyelid [27]. The cornea, the most anterior part of the eye, absorbs all wavelengths under 290 nm and 90% of wavelengths above 300 nm, protecting underlying structures from harmful UV rays [28–30]. We have recently demonstrated that absorption of UV rays induces CPD formation in the human cornea [31, 32]. Since UV light, a proven carcinogen in skin, generates promutagenic CPD lesions in the cornea, we find the low sensitivity of corneal epithelium to UV-induced cancer intriguing.

We have compared UV stress response in corneal epithelial cells (HCEC) and epidermal keratinocytes (NHEK). The probability that DNA damage leads to mutation depends on the frequency of its induction and the rate of its removal [33, 34]. Also, by eliminating damaged cells, apoptosis is an important antitumor mechanism [35, 36]. We thus analyzed these three important factors influencing UV-induced cellular transformation: UV-induced DNA damage frequency, repair and cell death sensitivity. In corneal and skin explants, we found greater CPD induction in the corneal epithelium, but a similar CPD levels in UV-exposed cultured NHEK and HCEC. We also found a 4 times faster repair of UV-induced CPD in HCEC when compared to NHEK. Since CPD are repaired by nucleotide excision repair a mechanisms in which DNA damage removal rate is highly dependent on its recognition, we have analysed the main recognition proteins, i.e. DDB2 and XPC, for transcript and protein levels as well as their localization following UV exposure. We have shown that corneal epithelial cells have more NER repair recognition proteins localized to the chromatin. On the other hand, we found a lower sensitivity of HCEC to UV-induced apoptosis when compared to NHEK. We have also measured p53 protein, an important actor that orchestrates the cell response to genotoxic stress. We have found an increased level of nuclear p53 in HCEC when compared to NHEK. The efficient repair might help protecting the corneal epithelium against UV-induced neoplasia. To our knowledge, this is the first study exposing differential efficiency towards UV stress response between cells of two distinct human tissues.

## Results

### Sensitivity to UV-induced DNA damage formation

We have previously shown that CPD are induced by each UV type in the anterior segment of the human eye [31, 32]. However, those results do not allow us to compare sensitivity of the corneal epithelium and the epidermis to UV-induced CPD formation. In order to make a direct comparison, human skin biopsies and corneas were irradiated with 2,000 J/m^2^ of UVB and CPD formation was revealed by quantitative immunofluorescence with an antibody raised against CPD, according to a previously established protocol [31, 32].

**Fig 1:**
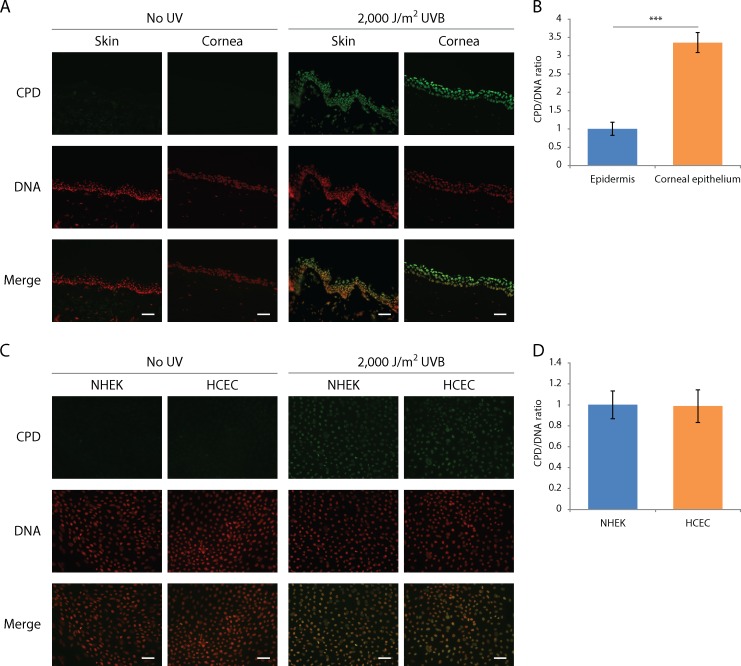
More CPD are found in corneal explants than in skin explants following a same dose of UV-irradiation but similar levels of CPD are detected in cultured NHEK and HCEC. UVB-induced CPD formation in skin and cornea was assessed in human skin biopsies, eyes and cultured NHEK and HCEC irradiated with 2,000 J/m^2^ of UVB. CPD were detected with an anti-CPD antibody (green) and DNA was counterstained with DAPI (red). (A) Immunohistofluorescence of UVB-induced CPD in skin and cornea shows an important induction of CPD in both epidermis and corneal epithelium. (B) Quantitative analysis of immunohistofluorescence. Relative CPD/DNA ratio from the epidermis and the corneal epithelium indicates that 3.4 times more CPD are found in the corneal epithelium from explants than in the epidermis from skin explants. (C) Immunocytofluorescence of UVB-induced CPD in cultured HCEC and NHEK. (D) Quantitative analysis of immunocytofluorescence. Relative CPD/DNA ratio from HCEC and NHEK show there is not difference in CPD induction between both cell types. Data information: (A, C) Scale bar, 50μm. (B, D) Data presented as means ± SEM. ***P < 0.001; Student’s ***t***-test. (A, B) Experiment was repeated on 5 skins (N = 5) and 4 corneas (N = 4) in duplicate (n = 2). (C, D) Experiment was repeated on 3 strains of NHEK and HCEC (N = 3).

No CPD were detected in unirradiated skin or corneal explants, indicating accumulated CPD from previous sunlight exposure were minimal (Fig 1A). UVB-induced CPD are found in both irradiated skin biopsies and corneal explants (Fig 1A). In accordance with what we previously observed, the quantity of CPD decreases with increasing depth in both tissues [31, 32]. It can be observed that CPD are more frequently formed in the corneal epithelium when compared to the epidermis (Fig 1A). The quantified CPD signal reveals that when irradiated with a given dose of UVB, 3.4 times more CPD are induced in the corneal epithelium than in the epidermis (Fig 1B). We have then analyzed CPD induction in cultured HCEC and NHEK and the level of UVB-induced CPD in both cell type is identical (Fig 1C and 1D).

### Sensitivity to UV-induced cell death

In response to the genotoxicity of UV-induced DNA damage, cells initiate different protection mechanisms. Unrepaired bulky DNA adducts lead to transcription or replication arrest [37] to which cells respond by triggering programmed cell death [38]. It is well defined that apoptosis, by eliminating cells containing large quantities of mutagenic DNA damage, is an efficient protection mechanism against UV-induced neoplasia [35, 36]. To investigate sensitivity to UV-induced cell death, cultured NHEK and HCEC were irradiated with 1,000 to 5,000 J/m^2^ of UVB. Cells were harvested 16h after irradiation and apoptosis and necrosis were assessed using annexin V FITC and propidium iodine (PI), analyzed by flow cytometry.

**Fig 2:**
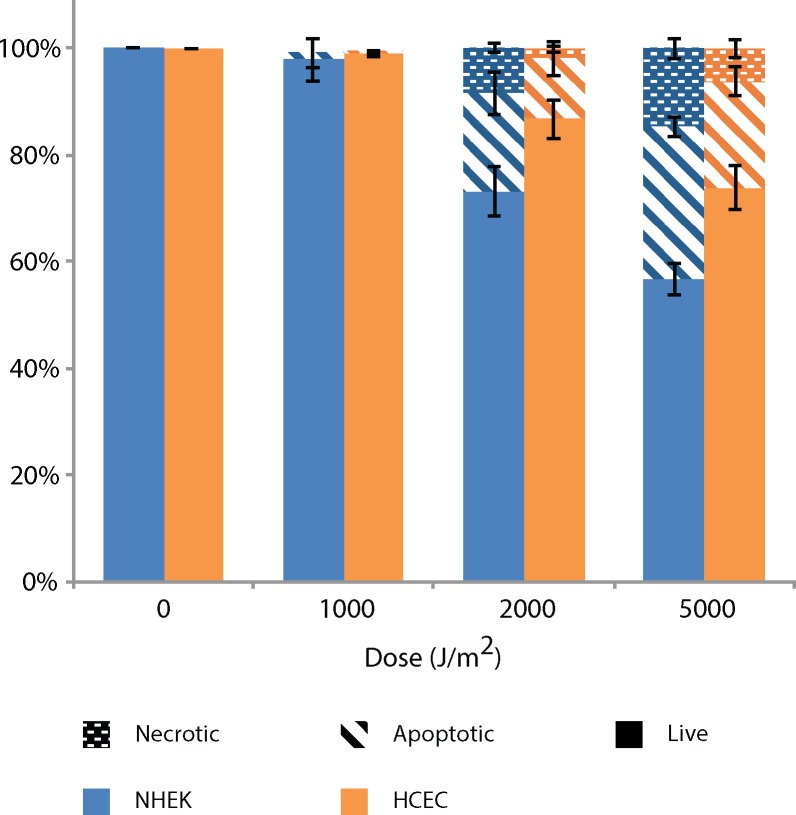
NHEK are more sensitive to UV-induced cell death when compared to HCEC. Apoptosis and necrosis is assessed by Annexin V/FITC and PI assay in UVB-irradiated NHEK and HCEC with doses ranging from 1,000 J/m^2^ to 5,000 J/m^2^ of UVB at 16h post-irradiation. Dotted, dashed and plain bars represent necrosis, apoptosis and live cells, respectively. Data information: Data presented as means ± SEM. Percentage of necrotic, apoptotic and live cells are significantly different (p<0.05) between NHEK and HCEC irradiated at 5,000 J/m^2^ UVB, according to the Student’s ***t***-test. Experiment was repeated on 3 NHEK cultures (N = 3) and 3 HCEC cultures (N = 3) in triplicate (n = 3).

We observed direct correlation between cell death and UVB dose (Fig 2). At 1,000 J/m^2^ of UVB, we detect no lethality in both cell types. At 2,000 J/m^2^ of UVB, cells mostly survived with a 27% death (18.5% apoptosis / 8.5% necrosis) and 13% death (11,25% apoptosis / 1.75% necrosis) for NHEK and HCEC, respectively. At 2,000 J/m^2^ of UVB, we notice a difference in cell survival between NHEK and HCEC, the later being less sensitive. However, this difference is non-significant. At 5,000 J/m^2^ of UVB, the cell sensitivity of NHEK (57% live / 28.5% apoptosis / 14.5% necrosis) is significantly higher than HCEC (74% live / 20% apoptosis / 6% necrosis). We found the NHEK to be more sensitive to UV-induced apoptosis and necrosis than HCEC.

### Efficiency of UV-induced DNA damage repair

If the amount of induced DNA damage is under the threshold triggering programmed cell death, specific repair mechanisms are prompted. In human, UV-induced bipyrimidine photoproducts (CPD and 6-4PP) are removed from DNA by nucleotide excision repair (NER) [39, 40]. Xeroderma pigmentosum (XP) patients, deficient in at least one protein of the NER pathway, show impaired repair of UV-induced DNA damage. The risk of these patients to develop skin cancer or neoplasia in the anterior part of the eye is estimated to be 2,000 fold greater than in the general population [41]. By maintaining DNA integrity from UV-irradiation, NER is a crucial anti-tumor mechanism in human. To assess efficiency of UV-induced DNA damage repair, cultured cells (NHEK and HCEC) and human corneal explants were irradiated with 400 J/m^2^ of UVB and put back in cell or organ culture conditions. From 0 to 48h post-irradiation, DNA was harvested from irradiated cells and remaining DNA photoproducts were visualized by immunoblotting technique using an anti-CPD or anti-6-4PP antibody.

**Fig 3:**
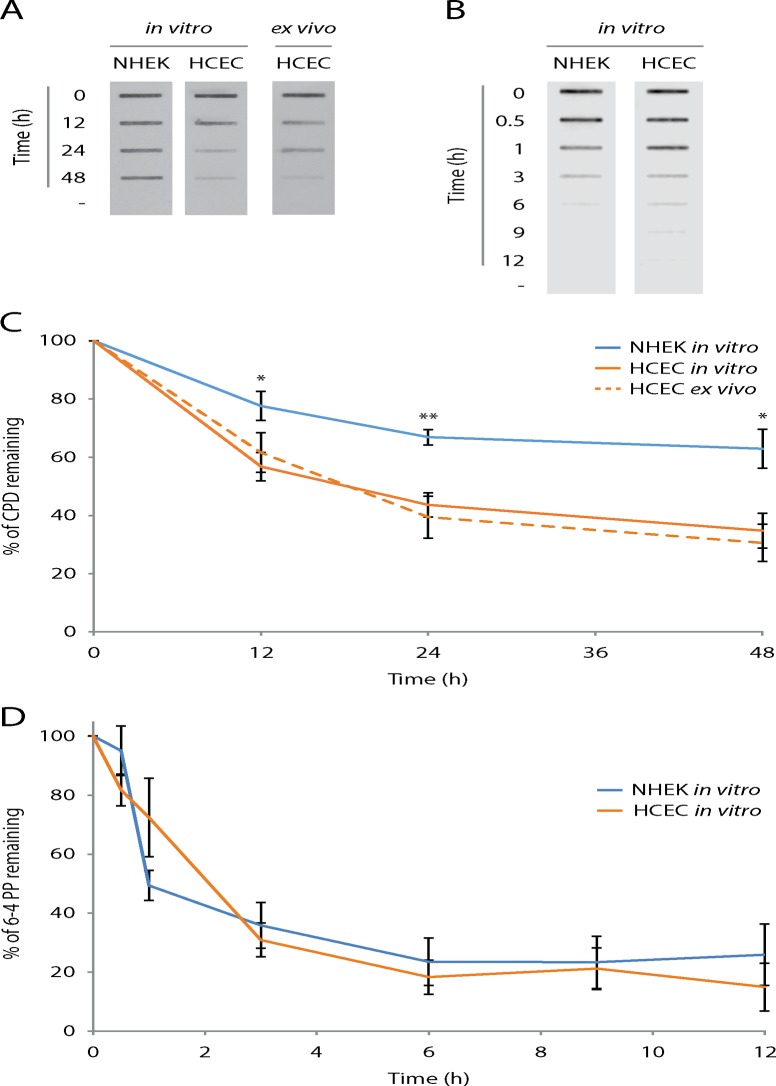
CPD repair, but not 6-4PP, is more efficiently in HCECN than NHEK. UVB-induced CPD and 6-4PP repair kinetics in NHEK and HCEC by DNA slot-blot. (A) and (B) Representative images of DNA slot-blots. DNA extracts from NHEK and HCEC cultures (*in vitro*) along with corneal epithelium (HCEC *ex vivo*) harvested before (-) and at different time points following a 400 J/m^2^ of UVB irradiation were blotted. UV-induced DNA damage was revealed with specific antibodies; (A) CPD and (B) 6-4PP. (C) and (D) Quantitative analysis of DNA slot-blots. Percentages of DNA damage remaining are calculated by relating the intensity of signals in the DNA of irradiated cells to the intensity of their unirradiated counterparts. The analysis shows that repair of CPD, but not 6-4PP, is significantly faster in HCEC when compared to NHEK. Data information: (C and D) Data presented as means ± SEM. (C) ***P***-values were calculated between NHEK *in vitro* and HCEC *in vitro* only. *P < 0.05, **P < 0.01; Student’s ***t***-test. (A and C) Experiment was repeated on 4 NHEK *in vitro* (N = 4), 7 HCEC *in vitro* (N = 7) and 9 HCEC *ex vivo* (N = 9) in duplicate (n = 2). (B and D) Experiment was repeated on 4 NHEK *in vitro* (N = 4) and 6 HCEC *in vitro* (N = 6) once (n = 1).

We observe faster CPD removal in cultured HCEC as opposed to NHEK (Fig 3A). The CPD repair kinetic in NHEK can be compared to what is found in the literature [39, 42–44]. These cells reach about 60% of CPD remaining in 48h (Fig 3C). Repair rate of HCEC is significantly faster than NHEK, reaching a similar percentage of CPD remaining (50%) in about 12h, 4 times faster than the later. Moreover, the repair rate of HCEC is significantly faster than NHEK at every time points analyzed (12, 24 and 48h). In *ex vivo* HCEC, from cultured whole corneas explants, the CPD repair kinetic is highly similar to what is found in cultured *in vitro* HCEC (Fig 3A and 3C).

We have then assessed the repair kinetic of 6-4PP, the second most abundant UV-induced bipyrimidine photoproduct. NHEK and HCEC cultures were irradiated with 400 J/m^2^ of UVB and incubated in culture condition for 0, 0.5, 1, 3, 6, 9 and 12h post irradiation. DNA was extracted from cells and the remaining 6-4PP were assessed by immunoblotting.

The repair rate of 6-4PP is virtually identical in NHEK and HCEC (Fig 3B and 3D). The removal of 6-4PP is relatively fast compared to CPD, which is consistent with what has been previously published [39]. More precisely, more than 80% of 6-4PP are removed 6h post-irradiation (Fig 3D). Repair kinetic of 6-4PP are in every point similar between NHEK and HCEC, no statistical difference were found. However, there might be a small to moderate difference in 6-4PP repair between both cell types that would be difficult to highlight because of the fast repair rate of 6-4PP and the intrinsic variability of the technique.

### Damage recognition proteins and p53

In human NER, DNA damage recognition can be accomplished in two different ways: through RNA Pol II stalling on actively transcribed genes (TCNER) or globally throughout the genome (GGNER).

Impairment of the TCNER pathway via a deficiency in at least one of its specific proteins does not lead to higher susceptibility to develop UV-induced skin or ocular cancer [45]. Conversely, impairment of the GGNER pathway via a deficiency in DNA damage recognition proteins XPC or DDB2 [46, 47], leads to an important increased risk for UV-related cancer. The effect is particularly high for patients bearing functional mutations in XPC [41, 48]. Indeed, binding of XPC to the damage site is an essential step for the recruitment of subsequent NER proteins. As for patients bearing mutations in XPE, the effect is milder [49]. This may be explained by the fact that even though DDB2 greatly enhances NER efficiency, it is not essential *per se* for DNA damage recognition in GGNER [50]. Moreover, gene coding for p53 is the most frequently mutated gene in skin cancer [10]. The implication of p53 in the cellular response to genotoxic stress is well demonstrated and is implicated in apoptosis and DNA repair [51–53].

**Fig 4:**
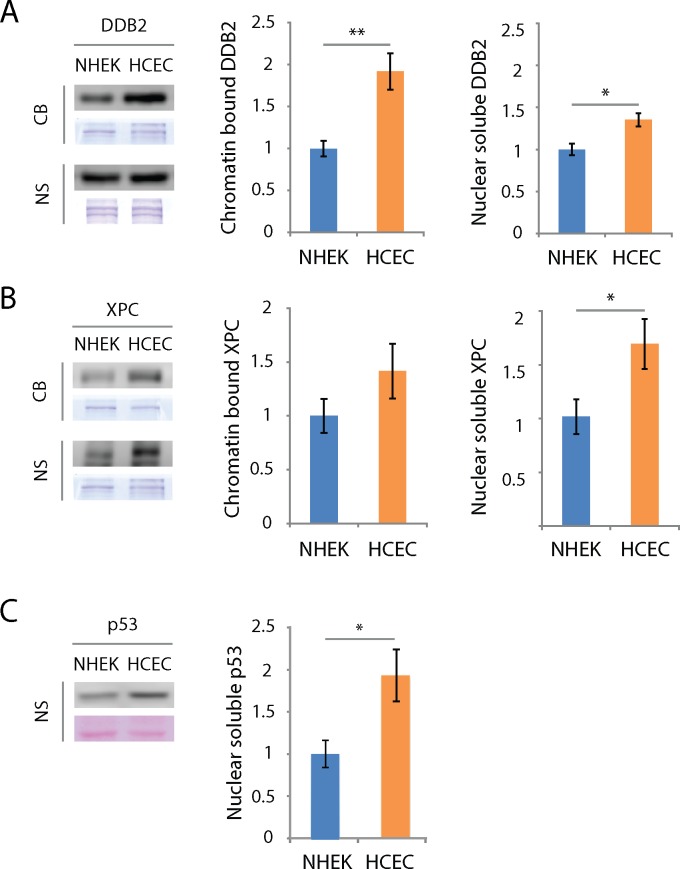
DDB2, XPC and p53 protein levels are higher in HCEC than NHEK. Representative Western immunoblots for DDB2 (A), XPC (B) and p53 (C) analysed in the chromatin bound (CB) and nuclear soluble (NS) protein extracts from NHEK and HCEC. Coomassie blue staining was used as a loading control. (A) The quantitative analysis of Western blots shows DDB2 protein levels higher in HCEC when compared to NHEK in both CB and NS fractions. (B) As for XPC, protein level is significantly higher in HCEC than NHEK only in the NS faction. (C) p53 level is significantly higher in NS fraction of HCEC when compared to NHEK. Relative HCEC protein levels are related to their NHEK protein levels counterparts. Data information: Data presented as means ± SEM. *P < 0.05, *P < 0.01; Student’s ***t***-test. (A to F) Experiment was repeated on 5 NHEK (N = 5) and 9 HCEC (N = 9) in triplicate (n = 3).

We thus investigated protein levels of DDB2, XPC and p53 in NHEK and HCEC. Proteins bound to the chromatin and soluble in the nucleus were extracted from cultured NHEK and HCEC and were used for western blot analyses with appropriate antibodies. DDB2 protein levels in the chromatin bound (CB) and nuclear soluble (NS) fractions are higher in HCEC when compared to NHEK (Fig 4A). More precisely, 1.9 and 1.4 times more DDB2 is found in the CB and NS fractions respectively of HCEC when compared to NHEK; these differences are significant (Fig 4A). XPC protein levels are also higher in both fractions of HCEC when compared to NHEK (Fig 4B). Quantification reveals more XPC in HCEC than NHEK, *i*.*e*. 1.4 times more in CB fraction and 1.7 times more in NS fraction (Fig 4B). However, the difference is significant only for the NS and not for the CB fraction. P53 protein levels are 1.9 times higher in NS fraction of HCEC when compared to NHEK (Fig 4C).

The mechanisms by which DDB2 and XPC recognise UV-induced DNA damage is well documented. DDB2 scavenges DNA until it recognises a distortion caused by a bulky adduct in the double helix [54]. Binding of DDB2 to DNA facilitates recruitment of XPC at damage site, but XPC has the capacity to bind DNA damage on its own [55, 56]. DDB2 is the substrate of a large complex, CRL4, which functions as an E3 ubiquitin ligase [57] [58]. For p53, under normal conditions, it is post-translationally stabilized in response to a variety of stress signals. This stabilization can then initiate different programs such as cell cycle arrest, senescence, or apoptosis [59]. The higher levels of DDB2, XPC and p53 in HCEC led us to hypothesize that the sequence of events after a UV-induced stress (i.e. translocation and degradation of DDB2 and XPC and stabilisation of p53 post-UV) may be modified in HCEC when compared to NHEK. To assess this, cultured HCEC and NHEK were irradiated with 400 J/m^2^ of UVB and proteins were harvested at different times (0, 0.25, 0.5, 1, 2, and 4h). Again, proteins were extracted from cell pellets, chromatin bound and nuclear soluble fractions were isolated and were used for western blot analyses. For each time point, the western blot signals for DDB2 and XPC were reported to the corresponding unirradiated control (NoUV), to which a value of 1 has then been attributed. The modified level (0-4h post-UV) compared to the basal level (NoUV) was then determined.

**Fig 5:**
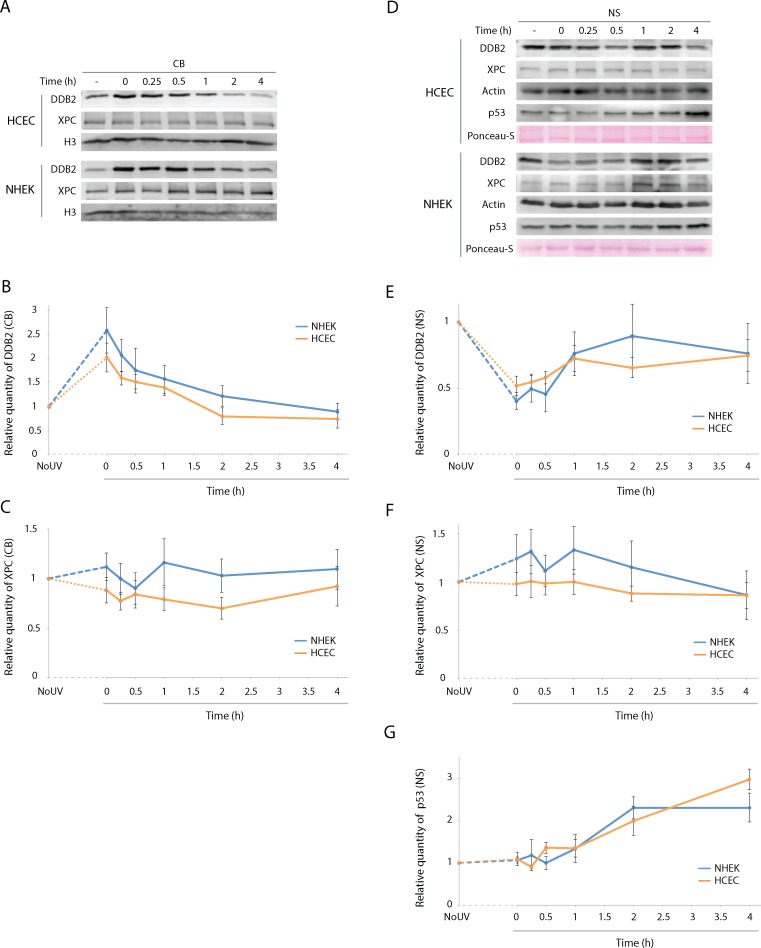
DDB2, XPC and p53 follow same induction/translocation pattern after UV exposure in NHEK and HCEC. Quantification and distribution of DDB2, XPC and p53 after UV exposure in NHEK and HCEC was assessed by Western immunoblot. (A) Representative image of Western immunoblots for DDB2 and XPC analysed from chromatin bound (CB) and for DDB2, XPC and p53 from nuclear soluble (NS) protein extracts of NHEK and HCEC cultures harvested before (-) and at different time points following a 400 J/m^2^ of UVB irradiation. (B) Quantitative analysis of Western blots shows a rapid increase of DDB2 on the CB fraction and a (E) rapid decrease in NS post-UV irradiation (unirradiated control vs 0h post-UV) in both NHEK and HCEC. This is followed by a partial or complete restoration of DDB2 level to the unirradiated control level in NS and CB fractions, respectively. (C) and (F) For XPC, the protein level varies to a lesser extent post-irradiation and was considered non-significant in both NHEK and HCEC. No significant difference in DDB2 and XPC levels modulation post-irradiation is observed between NHEK and HCEC. (G) For p53, the protein levels increase drastically 1h post-irradiation for at least 4h. Each western is quantified independently and is not compared with each other. The protein levels of DDB2, XPC or p53 post-irradiation are calculated as the percentage of variation in relation to the unirradiated level for each protein. A value of 1 is attributed to the NoUV for each protein and the post-irradiation levels are related to the NoUV. Data information: Data presented as means ± SEM. No significant difference was raised; Student’s ***t***-test. Experiment was repeated on 5 NHEK (N = 5) and 4 HCEC (N = 4) in duplicate (n = 2).

DDB2, XPC and p53 behave similarly in NHEK and HCEC following UVB-irradiation (Fig 5). More precisely, level of DDB2 in CB fraction is drastically augmented right after irradiation (0h), and gradually diminishes through time (Fig 5A). This drastic augmentation is most likely a redistribution of DDB2 from the NS to the CB than a rapid increase in protein synthesis. Signal quantification shows that, immediately after irradiation, DDB2 level increases to 2.6 and 2.0 times its initial level in NHEK and HCEC respectively (Fig 5B). DDB2 level then drops back to its initial level between 2 and 4h post-irradiation. Level of DDB2 in NS fraction is drastically lowered upon irradiation, and increases gradually afterwards (Fig 5D). Quantification of the signals shows that 0h after irradiation, DDB2 level falls approximately to half of its initial level in NHEK and HCEC, 0.52 and 0.40 respectively (Fig 5E). DDB2 never reached its initial level back during the analyzed period (up to 4h). In contrast, level of XPC in CB and NS does not vary significantly after UVB irradiation (Fig 5C and 5F). In accordance with what has been published previously, the level of NS p53 protein increases rapidly after the irradiation [60]. However, we found no difference in p53 induction post-UV between both cell types.

### DDB2 and XPC Transcript levels and protein half-lives

**Fig 6:**
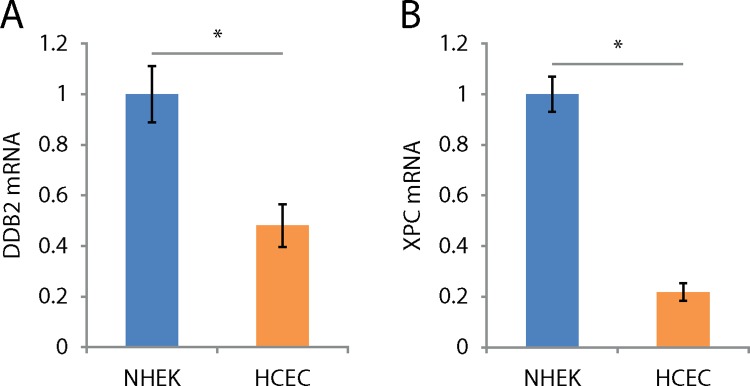
*DDB2* and *XPC* mRNA levels are lower in HCEC than NHEK. (A) and (B) Relative levels of *DDB2* and *XPC* mRNAs by q-RTPCR from NHEK and HCEC cultures. NHEK mRNA levels serve as baselines to which HCEC mRNA levels are related. The mRNA levels of *DDB2* and *XPC* are 2.1 and 4.6 times higher in NHEK when compared to HCEC, respectively. Data information: Data presented as means ± SEM. A fold change is considered significant when greater than 2 in q-RTPCR experiments. Experiment was repeated on 3 NHEK (N = 3) and 3 HCEC (N = 3) in quadruplicate (n = 4).

The amount of a given protein results from the balance between its synthesis and its degradation. Proteins are maintained at a certain level through regulation of transcription and protein half-life. To study the mechanisms by which HCEC show an increased amount of DDB2 and XPC, we first investigated the transcription level of coding genes for both proteins. Total mRNA was extracted from cultured NHEK and HCEC and used in quantitative real-time PCR analyses of *DDB2* and *XPC* mRNA levels. Q-RTPCR analysis revealed the mRNA levels of both *DDB2* and *XPC* are 2.1 and 4.6 fold lower in HCEC than NHEK, respectively (Fig 6A and 6B).

In response to its environment, the half-life of a protein may greatly fluctuate [61]. The incoherence between *DDB2* and *XPC* mRNA (Fig 6) and protein levels (Fig 4) suggests there is a difference in the half-life of these proteins. To assess half-lives of DDB2 and XPC proteins, NHEK and HCEC were cultured in the presence of cycloheximide, a compound known to prevent *de novo* protein synthesis. Cells were harvested after 0, 12, 24, 36 and 48h of culture in presence of cycloheximide, proteins were extracted and blotted to observe the rate of DDB2 and XPC protein degradation.

**Fig 7:**
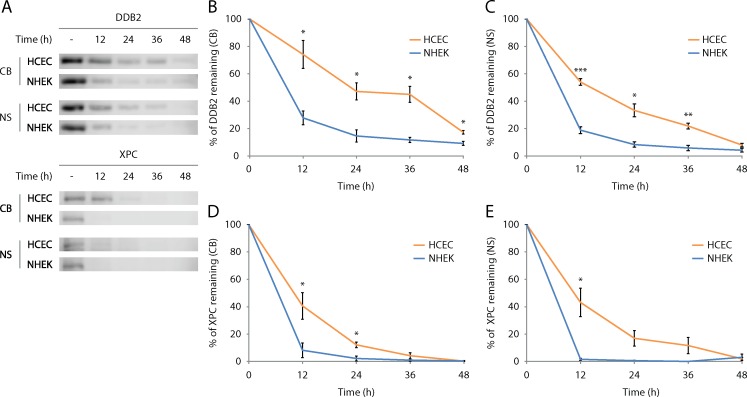
NER damage recognition proteins DDB2 and XPC are longer-lived in HCEC than NHEK. DDB2 and XPC protein half-lives in NHEK and HCEC were assessed by cycloheximide chasse assay. (A) Representative image of Western immunoblots of DDB2 and XPC analysed from chromatin bound (CB) and nuclear soluble (NS) fractions of NHEK and HCEC cultures without (-) and with added cycloheximide to the medium for different times to inhibit *de novo* protein synthesis. (B), (C), (D), and (E) Quantitative analysis of Western blots shows a decrease of CB and NS fractions of DDB2 and XPC over time post cycloheximide exposure in both NHEK and HCEC. However, the decrease is faster in NHEK for both CB and NS fractions when compared to HCEC, indicating a greater half-lives of DDB2 and XPC proteins in HCEC. Percentages of protein remaining are calculated by relating the intensity of signals from cells exposed to cycloheximide to the signals from their unexposed counterparts. Data information: Data presented as means ± SEM. *P < 0.05, **P < 0.01, ***P <0.001; Student’s ***t***-test. Experiment was repeated on 4 NHEK (N = 4) and 4 HCEC (N = 4) in duplicate (n = 2).

For both CB and NS fractions, most of DDB2 and XPC protein degradation occurs within 48h (Fig 7). DDB2 protein level in CB and NS fractions is more stable in HCEC when compared to NHEK (Fig 7B and 7C). More precisely, CB DDB2 protein half-life in HCEC is 22.3h whereas it is 8.3h in NHEK. In NS fraction, half-life of DDB2 protein in HCEC is estimated at 14.3h whereas it is at 7.4h in NHEK. The degradation of XPC in the CB and NS fractions is slower in HCEC when compared to NHEK, although the difference is less marked than for DDB2 protein (Fig 7D and 7E). Quantification reveals half-life of XPC of the CB fraction protein is estimated at 10.1h in HCEC and 6.5h in NHEK. In NS fraction, half-life of XPC is estimated at 10.6h in HCEC and 6.1h in NHEK.

## Discussion

### Sensitivity to UV-induced DNA damage formation

Although it has been previously described that ocular region would receive between 59% and 77% of UV light reaching the top of the head [62], UV light dosimetry of the eye is a controversial matter, as many influential factors (*e*.*g*. time of day, behaviour, and facial traits) cannot be correctly compensated. We thus want to acknowledge the fact that using same dose irradiation does not mimic natural exposure; the goal of this experiment was rather to assess sensitivity to UV-induced DNA damage induction in a controlled environment. In this manner, we found a greater sensitivity to CPD induction in corneal epithelium as opposed to the epidermis (Fig 1). Some physical differences between the cornea and the skin could explain the greater UV-sensitivity observed in the cornea. First, UV-shielding properties of their surface differ. Even if both are composed of a basal layer of cells, *stratum basale*, and overlying squamous cells, *stratum spinosum*, their thickness differ, *i*.*e*. 69 ± 4 μm and 53.4 ± 4.6 μm for epidermis and corneal epithelium, respectively [63]. Moreover, the 3 overlying strata of terminally differentiating cells (corneocytes) forming a hydrophobic lipid envelope of 26 ± 3 μm thick in the skin (*i*.*e*. *stratum corneum*, *stratum lucidum* and *stratum granulosum*) is absent in cornea. Wavelengths under 300 nm are partially filtered by those 3 overlying strata in skin [64]. In cornea, this is partially compensated by the lacrimal film, which potentially absorbs wavelengths up to 300 nm [65]. Since the irradiations were performed *ex vivo*, our model lacked the lacrimal film and its potential UV protection. We believe this may have led us to overestimate the amount of induced CPD in the cornea. Thus, even if we found a greater sensitivity to UV-induced CPD in the cornea, when compared to the skin, this result needs to be taken with caution.

Melanocytes play an important role in UV protection. The melanin they produce is distributed to adjacent keratinocytes cells in order to protect their nucleus against UV wavelengths [66]. In epidermis, melanocytes are distributed throughout the basal layer of cells [67], while in the cornea, they are only found in the periphery (*i*.*e*. the limbal palisades of Vogt) where they protect stem cells [68, 69]. This means that UV-filtering melanin can be found in all keratinocytes of the epidermis whereas it is absent in corneal epidermal cells [70]. When taken out of their *in vivo* environment, *in vitro* corneal (HCEC) and epidermal cells (NHEK) are equally sensitive to UV-induced CPD formation. This implies that the global context in which those cells are influences their sensitivity to UV-induced DNA damage formation.

### Sensitivity to UV-induced cell death

We have investigated UV-induced cell death sensitivity and our results show that NHEK are more sensitive to UV-induced apoptosis. This was at first surprising to us since up to 90% of UV-induced apoptosis signal comes from CPD [71, 72] and we found similar levels of UV-induced CPD in both cell types. However, the more efficient repair mechanism in HCEC might explain the lower sensitivity to UV-induced apoptosis. Indeed, we have found that CPD repair is much faster in the HCEC when compared to NHEK, even at the shorter time point analyzed. Since the role of apoptosis in cancer prevention is to eliminate damaged cells [35, 36], the fact that removal of CPD is faster might inhibit the apoptosis signal triggering. However, more work should be done to investigate this aspect.

### HCEC are more efficient to repair CPD

We showed a significantly more efficient CPD removal in HCEC than NHEK. We confirmed this result found with an *ex vivo* model, which is more representative of the multi layered cell environment of the corneal epithelium. We found no statistical difference between the repair kinetics of *in vitro* cultured HCEC and *ex vivo* corneal epithelium, confirming the validity of our *in vitro* cell culture model.

In order to confirm the efficient CPD repair in HCEC is due to a global increase of NER efficiency, we investigated repair of another bipyrimidine photoproduct, 6-4PP. In contrast with the CPD repair kinetics, we have found no difference in efficiency of 6-4PP repair between HCEC and NHEK. However, the fast rate of 6-4PP repair along with the limitations of the detection technique would make it difficult to find moderate differences in 6-4PP repair rates between both cell types. Thus, even if we have not seen any difference in 6-4PP repair rates between NHEK and HCEC, there might be a moderate difference we could not detect.

### DDB2, XPC and p53 in HCEC and NHEK

The link between carbons 6 and 4 of the two adjacent pyrimidines in a 6-4PP induces an important helix distortion of 44° [73]. This pronounced distortion makes them easily recognisable by XPC alone, which possess a 30-fold preference for 6-4PP over CPD [74]. On the contrary, the two links between corresponding carbons 5 and 6 of two adjacent pyrimidines, responsible for the CPD, cause a helix distortion of only 7° to 9° [75]. Given the weaker affinity of XPC towards CPD and the drastic acceleration of *in vivo* repair caused by the presence of DDB2 [56, 76, 77], it is believed CPD are first recognised by DDB2 which then recruits XPC to the damage site [55, 78]. In both nuclear and chromatin bound fractions, we found a significantly greater quantity of DDB2 in HCEC than NHEK (Fig 4). This result suggests the implication of DDB2 in the efficient repair of CPD in HCEC. However, many other factors could be involved and more work needs to be done. DDB2 is normally present on the chromatin, scavenging for the presence of DNA damage [54]. Our results show not only a greater presence of DDB2 in HCEC cells, but more precisely, this increase is particularly important on the chromatin bound fraction. It can be hypothesized that more DDB2 proteins actively scavenge DNA, thus allowing HCEC to react more promptly to UV stresses leading to the efficient CPD repair found in those cells. As for XPC, we found more XPC in HCEC than NHEK, but with a non-statistical difference in chromatin bound fraction and a barely significant one in the nuclear fraction. We showed an important translocation of DDB2 to the chromatin within the first hour after irradiation in both cell types. Xeroderma Pigmentosum patients harbouring defects in XPC and DDB2 genes are more prone to develop ocular surface (including the cornea) and skin cancers [79, 80], demonstrating the importance of DNA repair in cancer prevention of corneal cancer. [58, 81]

P53 is well known to be involved in the regulation of NER and apoptosis [51–53]. Moreover, p53 has been found to upregulate DDB2 and XPC at the RNA and protein levels [82]. We found a higher basal level of p53 in HCEC when compared to NHEK, which correlates with the fact that the level of DDB2 and XPC proteins is higher in HCEC. However, XPC and DDB2 mRNA levels were lower in HCEC than NHEK, which is contradictory with the high level of p53 in HCEC and with the finding of a higher DDB2 and XPC protein level in HCEC. On the other hand, we have found that DDB2 and XPC proteins have a greater half-life in HCEC than in NHEK. This would suggest that XPC and DDB2 increased levels in HCEC would rather be due to protein stabilization than an increase synthesis triggered by p53.

Taken together, our results suggest that the potential sensitivity of the corneal epithelium to UV-induced DNA damage might be palliated by efficient DNA damage repair [83–85]. In this study, we have compared the corneal epithelium and the skin epidermis, for genotoxic stress responses implicated in the early events of carcinogenesis: DNA damage induction by carcinogen and mechanisms of mutation avoidance (*i*.*e*. damage-repair and cell-death). We have shown even though the cornea appears very sensitive to UV-induced DNA damage, these cells seem to compensate by repairing CPD with high efficiency. The accrued levels of DDB2, XPC and p53 found in HCEC led us to hypothesize that those cells are better prepared to respond to a genotoxic insult. However, the level of DNA damage recognition protein and p53 is not the sole factor influencing the response to genotoxic stress and more experiments should be performed to determine if those proteins plays a crucial role in UV-induced stress response in HCEC.

In conclusion, to our knowledge, this study is the first to evaluate how corneal cells react to genotoxic stress, such as the one generated by exposure to UV radiation. Since UV-induced skin cancer is by far the most common cancer, many research have been conducted in order to understand how epidermal cells react to UV-induced genotoxic stress. However, since UV-induced ocular surface cancers are much less common in the corneal region, not many studies have been done in this regard. The fact that the repair of mutagenic CPD is much faster and the apoptosis is less efficient in cells of the corneal epithelium when compared to the skin epidermal cells will undoubtedly bring a lot of attention to this structure.

## Materials and Methods

All experiments performed in this study were conducted in accordance with our institution's guidelines and the Declaration of Helsinki. The research protocols received approval by the CHU de Québec institutional committee for the protection of human subjects and written consents to participate to the research project has been obtained and recorded according to the ethic committee recommendations.

### Organ and tissue processing

Human skin biopsies were obtained from faces (rhytidectomy) and breasts (mastectomy) of women ages 34 to 68. Whole human eyes were obtained from la Banque de tissus oculaires du CUO de Québec from men and women ages 51 to 84. Their corneas were mechanically excised for further analysis. Donors did not bear pathologies known to affect genotoxic stress response. Upon reception, samples were kept at 4°C until experimentation, at most 24h.

### Primary culture and UVB irradiation

Normal human epidermal keratinocytes (NHEK) were obtained from la Banque de cellules du LOEX. Human corneal epithelial cells (HCEC) were isolated from the corneal limbus of normal eyes obtained from la Banque de tissus oculaires du CUO de Québec following a procedure previously described [86]. Both primary cultures were plated on a feeder layer of irradiated murine Swiss-3T3 fibroblasts (ATCC, Rockville, MD) and grown under 5% CO_2_ at 37°C with medium changed every 2–3 days. Cells were cultured up to passage 5 prior to experimentation. Specimens (tissues or cells) were exposed to given UVB doses as described previously [31, 32].

### CPD Immunohistofluorescence of tissue samples

For irradiation of tissues *ex vivo*, skin biopsies were separated in two pieces. One was irradiated with 2,000 J/m^2^ of UVB while the other served as an unirradiated control. For each pair of human corneas, one cornea was irradiated and the contralateral cornea was used as an unirradiated control. CPD were revealed using immunohistofluorescence as described before [31, 32]. Revelation of CPD in irradiated cultured HCEC and NHEK was performed using the same fixation and immunofluorescence protocol as for the tissues specimens.[32]

Specimens were observed with Zeiss Axio Imager.Z2 microscope equipped with Zeiss AvioCam MRm camera. For each specimen, two distinct samples were observed on which two separate regions of about 450μm in length were photographed. CPD (damaged DNA) and DAPI (total DNA) signal quantification was made with the measuring module of Zeiss AxioVision Imaging System. A ‘‘Damaged DNA/Total DNA” ratio was derived from quantified signals to normalize CPD signal on total DNA quantity. A mean ratio was established by averaging the different results of a sole specimen, which was then used to establish an average ratio for the entire population of each tissue. 4 corneal and 5 skin explants were used and 3 cultures of NHEK and 3 of HCEC, all from different donors, were used. ***P***-values were derived from the two-tailed heteroscedastic Student’s ***t***-test.

### UV-induced cell death assay

Cells were all cultured to about 80% confluence to ensure similar growth rate. Cells were irradiated with doses ranging from 1,000 J/m2 to 5,000 J/m2 of UVB. Separate wells of cultured cells were kept unirradiated as controls. Cells were harvested 16h following UV irradiation and the analysis of cell viability, apoptosis and necrosis has been assessed using Vybrant 3 Annexin V / PI apoptosis kit (Molecular probes, Eugene, OR) as previously published [60]. This assay monitors the externalization of phosphatidylserine (PS) by annexin-FITC. In apoptotic cells, PS is translocated from the inner to the outer leaflet of the plasma membrane, thus exposing PS to the external cellular environment. Necrosis was monitored by staining nucleic acid using PI. The Annexin/PI stained cells were analyzed using a Becton-Dickinson FACS Accuri C6 flow cytometer on a two-color setting. NHEK cultures from 3 different donors and 3 HCEC cultures were used. ***P***-values were derived from the two-tailed heteroscedastic Student’s ***t***-test.

### UV-induced DNA damage repair assay

Cells were then irradiated in PBS with 400 J/m^2^ of UVB and incubated complete medium to be harvested in a timely manner dependant of the experiment. Dry cell pellets were kept at -80°C until further analysis. Total DNA was extracted from cultured NHEK and HCEC and directly from corneal epithelium (*ex vivo* HCEC) using a DNeasy Blood & Tissue Kit (QIAGEN) following the manufacturer’s protocol. For corneal explants, the central portion of the cornea (i.e. not including the limbus) was used. 400ng of DNA was denatured in 0.3N of NaOH incubated at 55°C for 1 hour. Immediately after incubation, ammonium acetate (Laboratoire MAT, Canada) was added in each sample in order to achieve a final concentration of 25μM. DNA was blotted on positively charged nitrocellulose membranes with a Bio-Dot® SF Microfiltration Apparatus (BIO-RAD, USA), and fixed by heat in an oven at 80°C for 3 hours. Membranes were then blocked for 1h in TBS with 5% nonfat dry milk.

Membranes were incubated overnight at 4°C with either a mouse anti-CPD (1:7,500; clone TDM-2, Cosmo Bio Co., Japan) or a mouse anti-6-4PP (1:2,000; clone 64M-2, Cosmo Bio Co., Japan) in TBS with 1% nonfat dry milk and 0.05% Tween® 20 solution. Every membranes were then incubated for 1h at room temperature with rabbit anti-mouse antibody conjugated to HRP diluted 1:5,000 (115-035-003, Jackson ImmunoResearch, USA) in TBS with 1% nonfat dry milk and 0.05% Tween® 20 solution. Analysis of membranes was performed with C-DiGit^®^ Blot Scanner (LI-COR Biosciences, USA).

Bound primary and secondary antibodies were then removed from membranes for 15 min with Pierce Restore™ PLUS Western Blot Stripping Buffer (Thermo Scientific, USA) and washed twice for 15min in TBS. Membranes were blocked and reprobed overnight at 4°C with a mouse anti-ssDNA monoclonal antibody diluted 1:1,000 (clone 16–19, EMD Millipore, Germany). Membranes were then washed, incubated with rabbit anti-mouse antibody conjugated to HRP (111-035-003, Jackson Immunoresearch, USA) and analyzed.

For CPD repair kinetic, NHEK cells from 4 different donors, HCEC cells from 7 donors and 9 *ex vivo* corneal epithelium were used. For 6-4PP repair kinetic, NHEK cells from 4 donors and HCEC cells from 6 donors were used. ***P***-values were derived from the two-tailed heteroscedastic Student’s **t**-test.

### RNA extraction and quantitative real-time PCR

Total RNA was extracted using TRIzol® Reagent (Life Technologies, Canada), quantity and quality was analyzed with 2,100 Bioanalyzer System (Agilent Technologies, USA) according the manufacturer’s protocol. Using the TaqMan^®^ Reverse Transcription Reagent (Applied Biosystems, Roche, Canada), 1μg of total RNA was used as template to synthesize cDNA, according to the manufacturer’s protocol. *DDB2* and *XPC* mRNA levels were quantitatively measured using the Rotor-Gene Q real-time thermocycler (Qiagen, Germany). Q-RTPCR reactions were achieved using the Brilliant III Ultra-Fast SYBR Green q-RTPCR Master Mix (Agilent Technologies, USA). The mix contained 5ng of cDNA, 20ng of primers and SYBR Green master mix (Agilent Technologies, USA). GAPDH has been used as endogenous control. All samples were made in quadruplicate for each gene. Our data analysis was based on the 2^-ΔΔCt^ method previously described [87].

The following primer sequences were used: for DDB2, forward *5’*CAT CAA AGG GAT TGG AGC TG*3’* and reverse *5’*CTA CTA GCA GAC ACA TCC AGG CTA*3’*. For XPC, forward *5*’TGA CCT CAG GGA CTT TCC AAG*3’* and reverse *5’*AAT TCT TAT CTC CAC TGG CTT CAG*3’*. For GAPDH, forward *5*’AAG GTC GGA GTC AAC GGA T*3’* and reverse *5*’GGA AGA TGG TGA TGG GAT TTC*3’*.

Unirradiated specimens served as calibrator and GAPDH as endogenous control. NHEK from 3 specimens and HCEC from 3 specimens were used. A fold change is considered significant when greater than 2 in q-RTPCR experiments.

### Cycloheximide chase assay

Cycloheximide (Sigma Aldrich, USA) was added to complete medium at a concentration of 50μg/mL. Chase experiment was started as soon as cycloheximide medium was added to cultures and cells were harvested 0, 12, 24, 36 and 48h post-treatment.

### Protein extraction and western blots

Cell pellets were resuspended in 2,5 volumes of cytosolic lysis buffer (10mM Tris pH 8, 0.34M Sucrose, 3mM CaCl_2_, 2mM MgCl_2_, 0.1mM EDTA, 1mM DTT, 0.5% NP40 and 40μl/mL protease inhibitor (Complete EDTA free, Roche, Canada) and kept on ice for 30min. After 2min of centrifugation at 1677g, the supernatant cytosolic protein fraction was removed and stored at -80°C. The remaining nuclear pellet was resuspended in 2,5 volumes of nuclear lysis buffer (20mM HEPES pH 7.9, 1.5mM MgCl_2_, 1mM EDTA, 150mM KCl, 0.1% NP40, 1mM DTT, 10% glycerol and 40μl/mL protease inhibitor) and broken using syringe and needle (21G). After 30min of centrifugation at 13,148g, the supernatant nuclear soluble protein fraction was removed and stored at -80°C. The remaining pellet was resuspended in 2 volumes of nuclease incubation buffer (20mM HEPES pH 7.9, 1.5mM MgCl_2_, 150mM KCl, 10% glycerol and 0.15 units/μl benzonase) and kept on ice for 1h. After 3min centrifugation at 13,148g, the supernatant chromatin bound fraction was removed and kept at -80°C. Cytosolic protein concentration was determined with the BCA kit (Thermo Scientific, USA) following the manufacturer’s protocol.

Protein extracts were separated on SDS-PAGE and blotted onto nitrocellulose membranes (BioTrace™ NT, PALL Life Sciences, USA). Proteins on blots were immunostained according to standard procedure. Primary antibodies used were: XPC polyclonal antibody diluted 1:500 (X1129, Sigma-Aldrich, USA), DDB2 polyclonal antibody diluted 1:500 (ab77765, Abcam, USA), H3 polyclonal antibody diluted 1:1,000 (ab1791, Abcam, USA), p53 monoclonal antibody diluted 1:250 (sc-126, Santa Cruz, USA) and actin polyclonal antibody diluted 1:1,000 (sc-1616, Santa Cruz, USA). Membranes were visualized using ECL (Western Sure Premium Chemiluminescent Substrat, LI-COR, USA) and the signal was quantified using C-DiGit® Blot Scanner (LI-COR Biosciences, USA).

For unirradiated cells protein assay, NHEK cells from 5 specimens and HCEC cells from 9 specimens were used. For UVB-irradiated cells protein assay, NHEK cells from 5 specimens and HCEC cells from 4 specimens were used. ***P***-values were derived from the two-tailed heteroscedastic Student’s **t**-test.
